# What determines patient preferences for treating low risk basal cell carcinoma when comparing surgery vs imiquimod? A discrete choice experiment survey from the SINS trial

**DOI:** 10.1186/1471-5945-12-19

**Published:** 2012-10-04

**Authors:** Michela Tinelli, Mara Ozolins, Fiona Bath-Hextall, Hywel C Williams

**Affiliations:** 1 Centre of Academic Primary Care and Health Economics Research Unit, University of Aberdeen, Aberdeen, UK; 2 Centre of Evidence Based Dermatology, University of Nottingham, A103, King’s Meadow Campus, Lenton Lane, Nottingham, NG7 2NR, UK

**Keywords:** Patient preferences, Discrete choice, Willingness to pay, Nodular and superficial basal cell carcinoma, Surgery, Imiquimod cream

## Abstract

**Background:**

The SINS trial (Controlled Clinical Trials ISRCTN48755084; Eudract No. 2004-004506-24) is a randomised controlled trial evaluating long term success of excisional surgery vs. imiquimod 5% cream for low risk nodular and superficial basal cell carcinoma (BCC). The trial included a discrete choice experiment questionnaire to explore patient preferences of a cream versus surgery for the treatment of their skin cancer.

**Methods:**

The self-completed questionnaire was administered at baseline to 183 participants, measuring patients’ strength of preferences when choosing either alternative ‘surgery’ or ‘imiquimod cream’ instead of a fixed ‘current situation’ option (of surgical excision as standard practice in UK). The treatments were described according to: cost, chance of complete clearance, side effects and appearance. Participants had to choose between various scenarios. Analysis was performed using a mixed logit model, which took into account the impact of previous BCC treatment and sample preference variability.

**Results:**

The analysis showed that respondents preferred ‘imiquimod cream’ to their ‘current situation’ or ‘surgery’, regardless of previous experience of BCC symptoms and treatment. Respondents were more likely to be worried about their cosmetic outcomes and side effects they might experience over and above their chance of clearance and cost. Those with *no experience* of surgery (compared *with experience*) valued more the choice of ‘imiquimod cream’ (£1013 vs £781). All treatment characteristics were significant determinants of treatment choice, and there was significant variability in the population preferences for all of them.

**Conclusions:**

Patients with BCC valued more ‘imiquimod cream’ than alternative ‘surgery’ options, and all treatment characteristics were important for their choice of care. Understanding how people with a BCC value alternative interventions may better inform the development of health care interventions.

## Background

To make an informed choice when choosing between surgery and imiquimod cream treatments for low risk basal cell carcinomas (BCCs) such as superficial BCC or small nodular BCC not located on the central face (as specified by the British Association of Dermatology BCC treatment guidelines [1]), patients need to balance trade-offs between different aspects (e.g. risk of scarring, clearance, and out-of-pocket costs) attached to these alternative treatments (Weston and Fitzgerald [2]; Essers et al. [3]). Our aim was to investigate patient preferences for ‘surgery’ or ‘imiquimod cream’ for the treatment of BCC using a discrete choice experiment (DCE; Ryan et al. [4]) approach in a sample of patients participating in a randomised controlled trial of surgery vs. imiquimod for low risk nodular and superficial BCC (Ozolins et al. [5]). We chose the Discrete Choice Experiments (DCEs) technique as it has been previously used in health care to evaluate different cancer screening strategies (Wordsworth et al. [6]; Marshall et al. [7]; Kruijshaar et al. [8]; van Dam et al. [9]; Hol et al. [10]); and willingness to pay (WTP) for methyl aminolevulinate photodynamic therapy vs surgery in BCC (Weston and Fitzgerald [2]) and for Mohs micrographic surgery vs surgery (Essers et al. [3]). Patient experience with previous treatment has been shown to influence the utility (benefit) patients derive from health care interventions (Salkeld et al. [11]; Ryan and Ubach [12]; Cheraghi-Sohi et al. [13]). We are also aware that information from such experiments can help health professionals to understand individual preferences for treatment, and, indeed, inform policy (Eberth et al. [14]; De Bekker-Grob et al. [15]). To our knowledge there is no evidence of research investigating: i) *how* patients with a BCC value alternative treatments on offer ii) the extent of preference variability or heterogeneity in the sample and iii) bearing in mind that BCCs are often asymptomatic and multiple, whether patients *with experience* of tumour symptoms and previous treatments may have better informed preferences. This study investigates how patients with a low risk BCC participating in a randomized controlled trial of excisional surgery versus topical imiquimod value the choice between the two treatment modalities.

## Methods

### The Discrete Choice Experiment (DCE) questionnaire

The Discrete Choice Experiment (DCE) technique is an attribute based approach that quantifies strength of patients’ preferences for the health care services or interventions. Respondents choose between alternative hypothetical interventions described in terms of their characteristics and associated levels. Results from the regression model identify which attributes, such as chance of clearance, side effects or convenience, are significant in the decision to choose, and their relative importance across treatments (i.e. magnitude of the attributes). Money as an attribute can also be used to estimate trade-off or Willingness To Pay (WTP, a monetary measure of benefit) for changes in attribute levels (e.g. WTP for improving by 1% their chance of clearance). The DCE technique, and its application to health care, is extensively discussed elsewhere (Ryan et al. [4]; de Bekker-Grob et al. [15]).

In this experiment, a list of attributes, their levels, and *status quo* alternative (see below) were derived from a previous DCE exercise applied to BCC (Weston et al. [2]), and from systematic discussion with the research team plus advice provided by a panel of experts. They were chosen to be general aspects of treatment that best described the alternative interventions on offer within the SINS trial i.e. ‘surgery’ vs. ‘imiquimod cream’ (Table 1). The “cost to you” attribute was added to find out how much patients valued the treatment. How the treatments and attributes were presented to the participants can be seen in the questionnaire (see Additional file 1).

**Table 1:** Summary of coding for discrete choice experiment attributes and their levels

**Attributes**	**Attribute levels**			**Variable names**
	***Surgery***	***Imiquimod cream***	***Current situation***	
1) Cost [cost to you]	£0	£0	£0	COST
	£150	£150		
	£300	£300		
	£500	£500		
	£750	£750		
2) Chance [Chance of complete clearance]	94%	50%	96%	CHANCE
	96%	70%		
	98%	90%		
3) Side effects	MILD	MILD	MILD	MILD SIDE EFFECTS
	(mild pain that does not disturb sleep)	(Mild irritation, burning or redness)	(mild pain that does not disturb sleep)	
	MODERATE	MODERATE		MODERATE SIDE EFFECTS
	(pain that sometimes might disturb sleep)	(Moderate irritation, burning, redness or weeping)		
	SEVERE	SEVERE		(compared with severe)
	(pain that disturbs sleep)	(Severe irritation, burning, redness or ulceration)		
4) Appearance	NORMAL	NORMAL	MODERATE CHANGE	NORMAL APPEARANCE
	(Barely visible scar)	(Not discoloured skin)	(noticeable scar)	
	MODERATE CHANGE	MODERATE CHANGE		MODERATE CHANGE IN APPEARANCE
	(Noticeable scar)	(Slight lightening skin)		
	SEVERE CHANGE	SEVERE CHANGE		(compared with severe change)
	(Slightly raised scar)	(Discoloured skin)		
**ALTERNATIVES**	SURGERY	IMIQUIMOD CREAM	CURRENT SITUATION	SURGERY
			(fixed surgery option from their experience alternative to an hypothetical ‘surgery’)	CREAM (compared with current situation)

**Figure 1:**
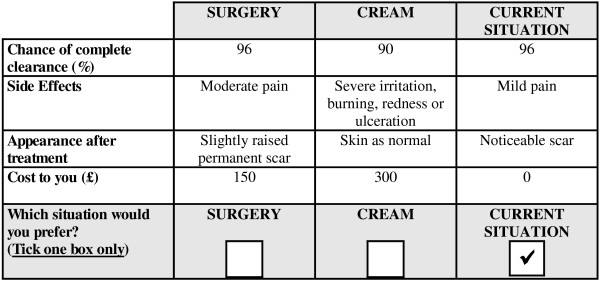
Example of Choice Set.

A DCE labelled (‘surgery’ vs. ‘imiquimod cream’) experiment was created from design catalogues [16] using a fold-over approach (Louviere et al. [17]; Rose and Bliemer [18]), as a commonly used technique to inform experimental design creation (de Bekker-Grob et al. [15]). Employing such a design minimised the number of choices for each respondent, from 135 (5^1^*3^3^) to 16 choices. To the 16-choice design, a third *status quo* alternative was added, representing surgical excision as current (standard) practice in UK hospitals (i.e. ‘current situation’). It defined an average surgical excision in a hospital setting with a 4 mm clear excision margin, use of a local anaesthetic injection, closure with sutures and removal of sutures on a separate visit. The ‘current situation’ was characterised by the following fixed levels: 96% chance of complete clearance; mild pain (that does not disturb sleep); a noticeable (but easy to cover) scar after treatment; and £0 cost to the patient (the procedure was undertaken free at the point of care in the UK National Health Service). An example of a choice is reported in Figure 1.

### Participants, sample size, setting and data collection

Our study sample included men and women of any age with low risk [1] nodular or superficial BCC participating in the SINS (Surgery vs Imiquimod in Nodular and Superficial basal cell carcinoma) study [5]. The SINS study is a randomized controlled trial to compare excision ‘surgery’ and ‘imiquimod cream’ for nodular and superficial basal cell carcinoma presenting in low risk areas. The study (ISRCTN48755084) received full ethical and hospital approval, and is conducted according to the declaration of Helsinki; all participants gave informed written consent.

Since size, type of experimental design, and the number of independent variables were unknown in advance, a minimum estimate of 50–100 responses per subgroup of interest was stipulated. The questionnaire was distributed to 183 of 501 consecutive patients participating in the trial (18^th^ to 200^th^), during their baseline visit (August 2003-January 2005). A minimum target response of 170 (response rate of 85%), allowing for comparison between two subgroups *with experience* and *with no experience* of BCC and treatment, was felt appropriate compared with similar studies (Pearmain et al. [19]).

### Analysis

Response rates, patient characteristics, and questionnaire completion: Descriptive statistics were presented as frequencies and percentages (binary variables) and median and inter-quartile range (continuous variables). Differences in patient characteristics between subgroups *with experience* and *with no experience* were reported and tested using the non parametric Mann–Whitney test and Chi square tests (where appropriate). SPSS software version 16 [20] was used to analyse the data.

Patient preferences: DCE modelling, its theoretical validity and subgroup analysis. Patient preference data were analysed using the mixed logit model (MLM, largely applied to multiple choice health care data, de Bekker-Grob et al. [15]). The Biogeme package [21] was used to support this analysis. Theoretical validity of responses was checked by testing the direction and significance of the model attributes. *A priori* we expected respondents to prefer: a decreased cost (a negative sign for ‘COST’); an increased chance of complete clearance (positive sign for ‘CHANCE’); a decreased severity of side effects (positive signs for ‘MILD SIDE EFFECTS’ and ‘MODERATE SIDE EFFECTS’); and improved appearance (positive signs for ‘NORMAL APPEARANCE’, and ‘MODERATE CHANGE IN APPEARANCE’). No *a-priori* assumptions were made concerning their preferred alternative (represented by the alternative specific constants (ASCs) for ‘surgery’ and ‘imiquimod cream’ options, ‘ASC_SURGERY_‘and ‘ASC_CREAM_’).

Subgroup analysis was undertaken to test whether different choices may result if respondents had experience of BCC and treatment. This follows current evidence in health care, reporting that respondents usually ascribe more value to the things they have experienced, (i.e. *status quo* bias: see Salkeld et al. [11]). The MLM model was presented considering all valid respondents (*whole sample*) and subgroups of those *with experience* and *with no experience* of BCC and treatment.

Estimated preferences were compared between experience subgroups. Difference in i) incremental WTP or ii) in proportion of respondents with preferences for a particular change in treatment characteristics (described by their mean and standard deviation) were reported and tested using independent *t* test statistics.

## Results and discussion

### Response rates, patient characteristics, and questionnaire completion

The DCE questionnaire was completed by 174/183 (95%) consecutive SINS trial participants. Overall 22% considered the questionnaire difficult/very difficult to complete (Table 2). The median age of the respondents was 65 years, 61% were men, 80% had a weekly income less than £300, and about 32% had previous experience of symptoms and treatment for BCC. Both subgroups *with experience* and *with no experience* presented socioeconomic characteristics similar to the *whole DCE sample* (See Table 2).

**Table 2:** **Patient characteristics (*whole sample *and subgroups relating to experience of BCC symptoms and treatment)**

	**Whole sample N=174**	**With no experience N=118**	**With experience N=56**	**With no vs. with experience**
	**% (n)**	**% (n)**	**% (n)**	**P value**
**Gender**				0.3
Male	60.9 (106)	63.6 (75)	55.4 (31)	
Female	39.1 (68)	36.4 (43)	44.6 (25)	
**Age**				0.1
Median [IQR]	65 [57–72]	63 [55–72]	68 [60–72]	
**Employment status**				N.A. ^1^
Employed	35.6 (62)	39.8 (47)	26.8 (15)	
Retired	63.2 (110)	59.3 (70)	71.4 (40)	
Unemployed	1.2 (2)	0.9 (1)	1.8 (1)	
**Weekly income**^**2**^				0.21
<£299	50.0 (80)	44.9 (53)	48.2 (27)	
£300-499	19.5 (34)	20.3 (24)	17.9 (10)	
£>=500	19.5 (34)	19.5 (23)	19.6 (11)	
(Not recorded to be excluded from analysis)	14.9 (26)	15.3 (18)	14.3 (8)	
**Previous experience of BCC symptoms and treatment**				
Yes	32.2 (56)	0 (0)	100 (56)	
No	67.8 (118)	100 (118)	0 (0)	
**Difficulties in completing the questionnaire**				N.A. ^1^
Extremely easy	13.2 (23)	9.3 (11)	21.4 (12)	
Easy	28.7 (50)	26.3 (31)	33.9 (19)	
Moderate	35.6 (62)	39.8 (47)	26.8 (15)	
Difficult	16.1 (28)	16.1 (19)	16.1 (9)	
Extremely difficult	6.3 (11)	8.5 (10)	1.8 (1)	

^1^ Cells have expected count less than 5. ^2^Some respondents presented not recorded data, as the question was not asked (dropped from assessments) or they did not complete it.

### Patient preferences

**Figure 2:**
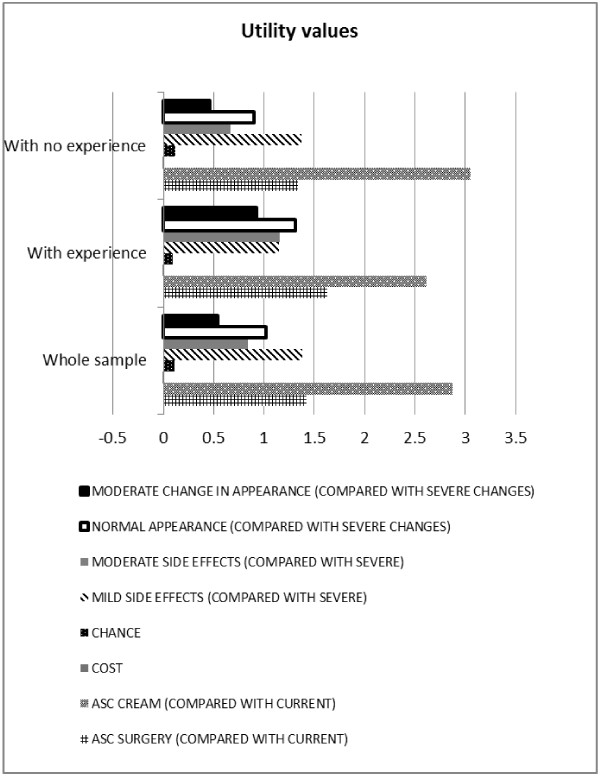
**Modelling patient preferences: Utility values.** Note: This Figure reports on average values only, whilst the complete output from the logistic regression model is presented in Tables 3 (*whole sample*) and 4 (subgroups *with experience* and *with no experience* of BCC symptoms and treatment). Alternative specific constants (ASC) for surgery (ASC _SURGERY_) and for cream (ASC _CREAM_) show the preferences of these alternatives relative to the current situation, everything else being equal. The cost attribute reported a mean value of −0.0031, and therefore it is less noticeable than the other attributes. Due to differences in scale factors across data sets, utility values from different subgroups are not directly comparable. For comparison between subgroups *with experience* and *with no experience* of BCC symptoms and treatment please see marginal WTP (Figure 3) and proportion of respondents (Figure 4). Overall findings from subgroup analyses are also presented in Table 4.

**Figure 3:**
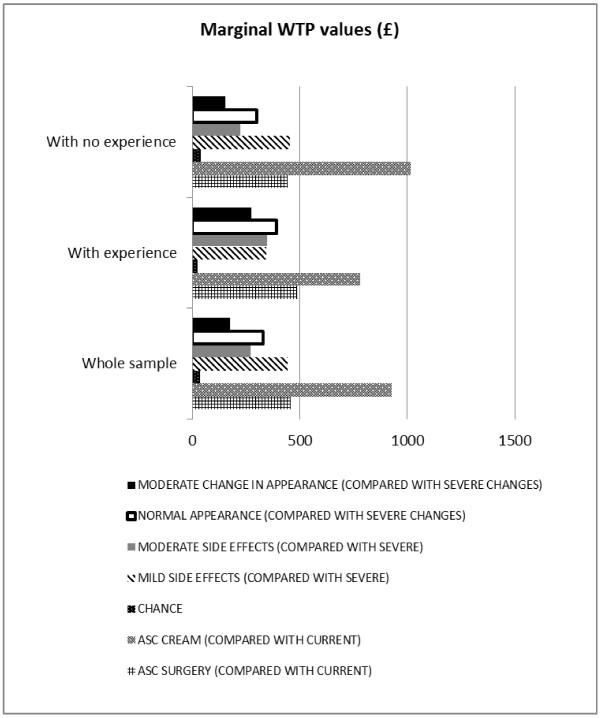
**Modelling patient preferences: Marginal WTP values (£).** Note: This Figure reports on mean values only, whilst the complete output from the logistic regression model, marginal WTP calculations (mean and standard deviation values), and their comparison between subgroups is presented in Tables 3 (*whole sample*) and 4 (subgroups with *experience* and *with no experience* of BCC symptoms and treatment). For ‘CHANCE’, ‘MODERATE SIDE EFFECTS’ and ‘NORMAL APPEARANCE’ differences in marginal WTP between subgroups (*with experience* vs. *with no experience* of BCC symptoms and treatment) are significant at the 95% level.

**Figure 4:**
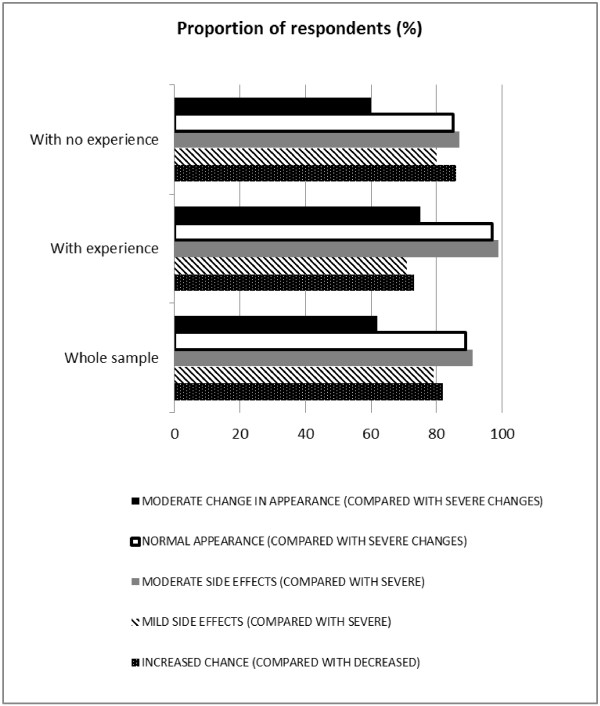
**Modelling patient preferences: the proportion of respondents (%) with positive effect of particular characteristic.** Note: The output from the logistic regression model used to inform these estimates is presented in Tables 3 (*whole sample*) and 4 (subgroups *with experience* and *with no experience* of BCC symptoms and treatment, and their comparison). For ‘MODERATE SIDE EFFECTS’ and ‘NORMAL APPEARANCE’ differences in preference distribution between subgroups (*with experience* vs. *with no experience* of BCC symptoms and treatment) are significant at the 95% level.

Specifications of the MLM model, its output for analysing the *whole sample* and subgroups with and without previous BCC are presented in Tables 3, 4. Figures 2, 3 display mean utility values and marginal WTP attached to the alternative treatments and their characteristics. The proportion of respondents for whom a particular characteristic had a positive effect is illustrated in Figure 4.

**Table 3:** **Patient preferences for the *whole sample *(n=174)**

		**Whole sample**				
		**Regression**^**1**^		**Incremental WTP**^**2**^**(£)**		**Their preference is for:**
		**Value**	***(SD)***	**Value**	***(SD)***	
**ASC**_**SURGERY**_	Coefficient	**1.4200**	*(0.253)*	**458.06**	*-*	Surgery (compared with current situation)
**ASC**_**CREAM**_	Coefficient	**2.8700**	*(0.398)*	**925.81**	*-*	Imiquimod cream (compared with current situation)
**COST**	Coefficient	**−0.0031**	*(0.000)*	*-*	*-*	Decreased cost
**CHANCE**	Mean	**0.1090**	*(0.018)*	**35.16**	*(38.71)*	increased chance, 82% of respondents
	SD	**0.1200**	*(0.022)*			
**MILD SIDE EFFECTS**	Mean	**1.3800**	*(0.314)*	**445.16**	*(541.94)*	mild side effects (compared with severe, 79% of respondents)
	SD	**1.6800**	*(0.282)*			
**MODERATE SIDE EFFECTS**	Mean	**0.8390**	*(0.265)*	**270.65**	*(199.68)*	moderate side effects (compared with severe, 91% of respondents)
	SD	**0.6190**	*(0.396)*			
**NORMAL APPEARANCE**	Mean	**1.0200**	*(0.265)*	**329.03**	*(267.74)*	normal appearance (compared with severe change, 89% of respondents)
	SD	**0.8300**	*(0.333)*			
**MODERATE CHANGE IN APPEARANCE**	Mean	**0.5400**	*(0.345)*	**174.19**	*(548.39)*	moderate change (compared with severe change, 62% of respondents)
	SD	**1.7000**	*(0.274)*			
**Number of respondents**		174				
**Number of observations**		2765 (=171 respondents *16 choices +1respondents*14 choices+1 respondent*8 choices+ 1 respondent*7 choices)				
**Log-likelihood**		−2013.9				
**Adjusted Rho-square**		0.333				
**LR statistic**		2047.47				

^1^The preferred model is a MLM and presents all characteristics (apart from ‘COST’, ‘ASC_SURGERY_’, ‘ASC_CREAM_’) as random and independently normally distributed. The simulation process is based on 500 draws. All coefficients (either fixed or random) and the standard deviations of the random coefficients are statistically significant at 95%. ^2^WTP estimates are based on the regression results in column 3 and rounded to 2 decimal places. The standard deviations are in parentheses. Marginal WTP for a unit change in a treatment characteristic = (mean coefficient of characteristic)/-(coefficient ‘COST’), with SD = (SD of characteristic coefficient)/-(coefficient ‘COST’).

Note: Alternative specific constants (ASC) for surgery (ASC _SURGERY_) and for cream (ASC _CREAM_) show the preferences of these alternatives relative to the current situation, everything else being equal.

*Key: SD = standard deviation, WTP = Willingness to Pay, LR = Likelihood Ratio*.

**Table 4:** Patient preferences according to experience of BCC and treatment

		**With experience**					**With no experience**				
		**Regression**^**1**^		**Incremental WTP (£)**		**Their preference is for:**	**Regression**^**1**^		**Incremental WTP (£)**		**Their preferences is for:**
		**Value**	***(SD)***	**Value**	***(SD)***		**Value**	***(SD)***	**Value**	***(SD)***	
**ASC**_**SURGERY**_	Coefficient	**1.6300**	*(0.457)*	**488.02**	*-*	Surgery (compared with current situation)	**1.3400**	*(0.310)*	**445.18**	*-*	Surgery (compared with current situation)
**ASC**_**CREAM**_	Coefficient	**2.6100**	*(0.719)*	**781.44**	*-*	Imiquimod cream (compared with current situation)	**3.0500**	*(0.488)*	**1013.29**	*-*	Imiquimod cream (compared with current situation)
**COST**	Coefficient	**−0.0033**	*(0.001)*			Decreased cost	**−0.0030**	*(0.001)*			Decreased cost
**CHANCE**^2^	Mean	**0.0898**	*(0.033)*	**26.89**	*(44.61)*	Increased chance (compared with deceased; 73% of respondents)	**0.1210**	*(0.026)*	**40.20**	*(37.54)*	Increased chance (compared with decreased; 86% of respondents)
	SD	**0.1490**	*(0.058)*				**0.1130**	*(0.023)*			
**MILD SIDE EFFECTS**	Mean	**1.1500**	*(0.553)*	**344.31**	*(616.77)*	Mild side effects (compared with severe; 71% of respondents)	**1.3700**	*(0.390)*	**455.15**	*(541.53)*	Mild side effects (compared with severe; 80% of respondents)
	SD	**2.0600**	*(0.474)*				**1.6300**	*(0.327)*			
**MODERATE SIDE EFFECTS**^2–3^	Mean	**1.1600**	*(0.480)*	**347.31**	*(−153.89)*	Moderate side effects (compared with severe; 99% of respondents)	**0.6710**	*(0.312)*	**222.92**	*(−199.67)*	Moderate side effects (compared with severe; 87% of respondents)
	SD	**0.5140**	*(0.519)*				**0.6010**	*(0.423)*			
**NORMAL APPEARANCE**^2-3^	Mean	**1.3100**	*(0.531)*	**392.22**	*(377.25)*	Normal appearance (compared with severe change; 97% of respondents)	**0.9000**	*(0.304)*	**299.00**	*(158.80)*	Normal appearance (compared with severe change, 85% of respondents)
	SD	**1.2600**	*(0.633)*				**0.4780**	*(0.498)*			
**MODERATE CHANGE IN APPEARANCE**	Mean	**0.9230**	*(0.564)*	**276.35**	*(407.19)*	Moderate change (compared with severe; 75% of respondents)	**0.4630**	*(0.433)*	**153.82**	*(634.55)*	Moderate change (compared with severe; 60% of respondents)
	SD	**1.3600**	*(0.539)*				**1.9100**	*(0.408)*			
**No. respondents**		56					118				
**No. observations**		896 (=56 respondents*16 choices)					1869 (=115 respondents*16 choices+ 1 respondent*14 choices+1 respondent* 8 choices+1 respondent*7 choices)				
**Log-likelihood**		−649.27					−1350.8				
**Adj Rho-square**		0.327					0.336				
**LR statistic**		670.18					1405.08				

Note: Alternative specific constants (ASC) for surgery (ASC_SURGERY_) and for cream (ASC_CREAM_) show the preferences of these alternatives relative to the current situation, everything else being equal. WTP estimates are based on the regression results in column 3 and rounded to 2 decimal places. The standard deviations are in parentheses. ^1^ The preferred model is a MLM and presents all characteristics (apart from ‘COST’, ‘ASC_SURGERY_’, ‘ASC_CREAM_’) as random and independently normally distributed. The simulation process is based on 500 draws. All coefficients (either fixed or random) and the standard deviations of the random coefficients are statistically significant at 95% ^2^ Differences in marginal WTP between groups are significant at the 95% level. ^3^Differences in preference distribution between groups are significant at the 99% level.

*Key: SD = standard deviation, WTP = Willingness to Pay, LR = Likelihood Ratio,* No. = Number of.

In the *whole group* and subgroups with and without previous BCC, all treatment choice attributes are statistically significant at 95% and therefore significant in the decision to choose. There is also evidence that individuals valued these characteristics differently. Overall, all characteristics have the expected directions supporting theoretical validity of the findings (see above). The positive signs on the alternative specific constants (ASCs) indicate that, everything else being equal, respondents preferred alternative treatments to their ‘current situation’, and, when choosing an alternative to their ‘current situation’, the ‘imiquimod cream’ was preferred overall to the ‘surgery’ (as indicated by the higher positive value). See Tables 3, 4 for further details.

#### Whole sample

Across all treatment characteristics choosing mild instead of severe side effects was the most valued aspect (as indicated by the largest significant coefficient of ‘MILD SIDE EFFECTS’ with a mean value of 1.38), whilst an increase in cost was the least preferred (‘COST’ with a mean value of −0.0031; see Figure 2).

Figure 3 and Table 3 also show respondents’ marginal WTP for a unit change in each treatment characteristic. For example, marginal WTP for choosing mild instead of severe side effects is £445.16 (standard deviation £541.94).

The proportion of respondents for whom a particular characteristic had a positive effect is reported in Figure 4, Table 3. For example, 79% of the sample preferred a change from severe to mild side effects, while the others did not value this change so highly.

#### Subgroup with experience of BCC and treatment

For those with previous BCC experience, the movement from severe change to normal appearance was the most important marginal change in an attribute (having the largest significant coefficient with a mean value of 1.31), and ‘COST’ remained the least preferred characteristic (see Figure 2, Table 4). The marginal WTP for choosing normal instead of severe change to appearance is £392.22 (standard deviation £377.25; Figure 3, Table 4). Ninety seven percent of the sample preferred normal appearance where as the others did not value this change so highly (Figure 4, Table 4).

#### Subgroup with no experience of a previous BCC and treatment

With *no BCC experience*, the movement from severe to mild side effects was the most important marginal change in an attribute (having largest significant coefficient with a mean value of 1.37), and ‘COST’ remained the least preferred characteristic (see Figure 2, Table 4). The marginal WTP when choosing mild instead of severe side effects was £455.15 (Figure 3, Table 4). Of the sample 80% preferred mild instead of severe side effects, while the others did not value this change so highly (Figure 1, Table 4).

#### Comparison between subgroups

Respondents preferred a change from the ‘current situation’ to alternative treatments and they mostly preferred moving to the ‘imiquimod cream’, regardless of their experience of a previous BCC and treatment. The subgroup with *no experience* valued more such change compared with the subgroup *with experience* (‘ASC_CREAM_’; £1013 vs. £781). The subgroup with *no experience* valued best choosing mild instead of severe side effects, and their willingness to pay for this choice was similar to the other subgroup (‘MILD SIDE EFFECTS’; £455 vs. £344, p=0.23). In the subgroups *with experience* the most valued characteristic was an improvement in their appearance from severe changes to normal appearance and they valued it more than the subgroup *with no experience* (‘NORMAL APPEARANCE’; £392 vs. £299, p=0.02). More details are presented in Figure 3 and Table 4.

When considering the difference in preference distribution across groups a greater proportion of participants *with experience* compared *with no experience* preferred the idea of a change from severe to moderate side effects (‘MODERATE SIDE EFFECTS’; 99% vs. 87%, p<0.01), or from severe change to normal appearance (‘NORMAL APPEARANCE’; 97% vs. 85%, p<0.01; see Table 4). Other random coefficient distributions did not present statistically significant differences between groups (‘CHANCE’ p=0.13; ‘MILD SIDE EFFECTS’ p=0.45; ‘MODERATE CHANGE IN APPEARANCE’ p=0.11).

### Summary of preference findings

This Discrete Choice Experiment (DCE) exercise was embedded within a wider randomised controlled trial. This provided the main trial with new empirical evidence of strength of patient preferences for alternative treatments on offer for low risk BCC, the impact of previous experience of a BCC and treatment on their preferences, and a measure of the spread or heterogeneity of preferences in the sample.

To our surprise, respondents preferred the ‘imiquimod cream’ treatment to their ‘current situation’ or alternative ‘surgery’, regardless of their experience of a previous BCC and treatment, though it might be questioned whether respondents *with no experience* of a condition or treatment are in a position to inform decision making (Gafni [22]).

Our study showed that regardless of their experience, respondents valued all aspects of treatment, but they were more likely to be worried about their cosmetic outcomes and side effects they might experience over and above their chance of clearance and cost. A cream option with better cosmetic outcomes was more appealing to them than a surgery intervention with better clearance outcomes.

Respondents *with experience* of a previous BCC and treatment (usually surgical) valued the ‘imiquimod cream’ option less than those without such experience, a finding supported in the literature of *status quo* bias, where people are more likely to adopt a conservative response to health services innovations (Salkeld et al. [11]; Ryan and Ubach [12]; Tinelli et al. [23]). In this particular case respondents’ experience for a surgery intervention might have limited their shift of preferences from their *status quo* to an innovative ‘imiquimod cream’ intervention on offer.

With experience of a previous BCC and surgical intervention (with risk of permanent scar), the movement from severe change to normal appearance was the most important marginal change; this subgroup were more likely to value a treatment with the best cosmetic outcomes, regardless of other characteristics, compared with those with no previous BCC.

### How people differed

Evidence of significant variation (heterogeneity) of preferences was found in the chance of clearance, side effects and appearance characteristics. Preferences for treatment characteristics were not specific to one particular improvement in their cosmetic outcomes or chance of clearance, though some changes might be regarded as not sufficient e.g. only 62% preferring moderate change vs. severe change. The distributions of preference were statistically different across experience subgroups. Results confirmed that respondents with experience of a BCC were more likely to value a treatment with the best cosmetic outcomes, whilst respondents with no past BCC might value less highly such improved characteristics.

### Study strengths and limitations

A particular strength was using a multiple choice design with inclusion of a *status quo* option; forcing a choice i.e. no *status quo* alternative when this reflects the reality may result in an overestimation of responses (Boyle et al. [24]).

Other strengths included exploring heterogeneity of responses, and the mixed logit model (MLM) allowed statistical investigation of how preferences varied across groups. Information on variation (heterogeneity) of preferences is recognised as an important aspect when integrating patient views into decision making (de Bekker-Grob et al. [15]). A MLM was applied to the data as it is commonly used to analyse multiple choice health care data (de Bekker-Grob et al. [15]), although alternative models, such as the latent class model, could also be adopted (Hensher et al. [25] Hensher and Greene [26]). Our modelling study also confirmed the importance of exploring preference variation across groups to better understand and implement innovative services on offer. Patient knowledge of the condition and treatment experience might provide more informed choice.

A limitation is that participants in this study are unlikely to have experienced topical imiquimod, thus we could not assess how previous imiquimod experience influenced their preferences (including the risk of possible reactions/side effects to the treatment), whereas we could at least partly with surgical excision as some had already experienced it for a previous BCC. It would have been informative to analyse the DCE further, for example, comparing any changes in patient preferences later in the study after experiencing surgery or imiquimod cream. Also, patients with both nodular and superficial BCC were included in the current analysis. Future studies could aim to collect larger sample sizes to perform a subgroup analysis according to the type of BCC affecting the patients. Time constraints did not allow us to further inform the decision on the choice of the attributes and their levels with patient interviews and focus groups. Other attribute levels (e.g. different levels for the cost attributes pending on the specific treatment compared) could be considered for future exercises and informed by trial economic evaluation.

Although our 95% overall response rate was high, respondents in this DCE survey might not have been representative of all BCC patients because they are all participants in the SINS trial, who by definition are more equipoise and willing to consider both options of ‘imiquimod cream’ and alternative ‘surgery’ intervention; also it is not a random sample. Most patients (with or without previous surgical experience) who had a strong preference for a *status quo* approach with surgical excision would probably not have chosen to participate in the SINS trial, and therefore their preferences were not captured by this DCE survey. Of those choosing not to take part in the study 48% (126/265) gave the reason as wanting to have surgery. A further seven dropped out of the study after randomisation because they did not want surgery.

The DCE can provide very useful information, but the completion difficulties and time taken in this elderly population (median age 68, up to a maximum of 92 years) are not to be underestimated – many found it difficult to understand the concept of choosing between hypothetical situations and hence took up to an hour to complete. The research nurse helped them to understand what they had to do. The results showed, however, consistency of answers, suggesting that participants were not just making random choices. DCE surveys will benefit from providing participants with help and support from experienced research staff during questionnaire completion if needed.

Although this research refers to a representative group of mainly elderly patients in the UK with low risk BCC, our findings of attitudes and preference to treatment options may not be generalisable to other countries and ethnic groups. It is also important to emphasise the low risk nature of this BCC population. The trade-off between clearance and cosmetic results in recurrences of aggressive tumours for example is likely to be different from those observed in this study population.

## Conclusions

Understanding how people with BCC value alternative interventions using the DCE technique may better inform the development of health care interventions, although this particular application proved data collection to be challenging and time consuming; elderly participants are likely to need help and support.

## Abbreviations

ASC: Alternative specific constant; BCC: Basal cell carcinoma; DCE: Discrete choice experiment; MLM: Mixed logit model; SINS study: Surgery vs. imiquimod in nodular and superficial basal cell carcinoma; WTP: Willingness to pay; vs: Versus or compared with.

## Competing interests

The authors declare they have no competing interests.

## Authors’ contributions

MT designed the discrete choice experiment, analysed the data, wrote the first draft of the manuscript, and revised the manuscript. MO provided input to the design of the experiment, entered and processed the data, revised the manuscript, and coordinated submission. FB-H provided input to the design of the experiment, and revised the manuscript. HCW provided input to the design of the experiment, and revised the manuscript. All authors read and approved the final manuscript.

## Pre-publication history

The pre-publication history for this paper can be accessed here:

http://www.biomedcentral.com/1471-5945/12/19/prepub

## Supplementary Material

Additional file 1 “**Questionnaire for SINS study”.** Contains the questionnaire used to collect DCE responses from each respondent. Click here for file
