# Supplementary material for: What determines patient preferences for treating low risk basal cell carcinoma when comparing surgery vs imiquimod? A discrete choice experiment survey from the SINS trial
Source: BMC Dermatol. 2012 Oct 4;12:19. doi: 10.1186/1471-5945-12-19 (PMC3532314; doi:10.1186/1471-5945-12-19)
Supplement: Additional file 1 — “Questionnaire for SINS study”. Contains the questionnaire used to collect DCE responses from each respondent. [file 1471-5945-12-19-S1.doc]

# Questionnaire for SINS study

***Section 1: Your treatment***

#

# We would like you to consider the following 2 treatments of your BCC:

**1. SURGERY –** surgical removal of the BCC. It is a **once only** treatment that requires attendance **at the hospital** outpatients department.

**2. CREAM** – application of a cream to the BCC. It is applied by you, **at home**, once a day for a period of **6 to 12 weeks**.

The treatment received could vary according to:

- **The chance of complete clearance.** This means the probability that BCC is completely removed.
  - **Example:** a 70% chance would mean that 70 people have BCC completely removed after the treatment out of every 100
- **Side-effects**. It may be possible to experience some unpleasant effects due to the treatment.
  - **Surgery:** may be painful
  - **Cream:** Is likely to cause irritation, redness or burning during treatment
- **Appearance after the treatment.** After the treatment the skin may not be completely normal.
  - **Surgery:** may leave some scarring on the skin
  - **Cream:** Does not usually cause scaring. It is possible to have slight lightening or discoloured/bleached skin around the treated area
- **Cost to you.** Here we are concerned with **how much you value the treatment**. One way to find out your value is to ask how much you are willing to pay. You will be asked to imagine 16 hypothetical scenarios where you must pay for treatments out of your own pocket. We must stress that this is **only a valuation exercise** and these treatments will continue to be provided **free of charge on the NHS**.

***Remember that if you spend money on treatment you will not have this to spend on other things!***

## PLEASE NOTE: *IF THE CREAM FAILS TO WORK YOU WILL REQUIRE SURGERY AND IF THE SURGERY FAILS TO WORK YOU WILL REQUIRE FURTHER SURGERY*.

# We would like to include your preferences between the 2 alternative treatments presented before compared to the current situation.

***Section 2: What would you choose?***

**CURRENT SITUATION**

**A once only SURGERY treatment that requires attendance at the hospital outpatients department.** It is currently described as follows:

**Chance of complete clearance 96 %**

**Side Effects Mild pain**

**Appearance after treatment Noticeable but easy to cover permanent scar**

**Cost to you £ 0**

# We would like you to answer the following 16 scenarios as accurately as possible.

Please see **the example** in the next page, which will help you fill in the following scenarios.

**EXAMPLE**

|  | ***SURGERY*** | ***CREAM*** | ***CURRENT SITUATION*** |
| --- | --- | --- | --- |
| ***Chance of complete clearance (%)*** | **94** | **90** | **96** |
| ***Side Effects*** | **Moderate pain** | **Mild irritation, burning or redness** | **Mild pain** |
| ***Appearance after treatment*** | **Slightly raised permanent scar** | **Skin as normal** | **Noticeable but easy to cover permanent scar** |
| ***Cost to you (£)*** | **300** | **150** | **0** |
| ***Which situation would you prefer?***  ***(Tick one box only)*** | ***SURGERY*** | ***CREAM*** | *CURRENT SITUATION* |

In this case **you prefer**:

- **A 90% chance of complete clearance;**
- **Mild irritation, burning or redness as possible side effects;**
- **Skin as normal after the treatment;**
- **A cost to you of £150.**

Rather than:

- An over 94% chance of complete clearance;
- Mild/Moderate pain as possible side effects;
- Easy to cover/slightly raised permanent scar after the treatment;
- A cost to you up to £300.

## YOUR PREFERENCES

## Please answer every question, completing only one box for each scenario. There is no right or wrong answer. It is only your personal opinion that matters. Assume other factors about treatment are the same.

## Scenario 1

You are told that :

- The chance of complete clearance of your BCC will be 96% if you have a surgery and 90% if you apply the cream.
- You will experience pain that disturbs sleep for a time following surgery and mild irritation, burning or redness DURING the treatment period if you apply a cream.
- Following treatment you will have a noticeable but easy to cover scar if you have surgery and discoloured or bleached skin around the treated area if you apply the cream
- The cost to you would be £150 for the surgery and £300 for the cream.

Summary

|  | ***SURGERY*** | ***CREAM*** | ***CURRENT SITUATION*** |
| --- | --- | --- | --- |
| ***Chance of complete clearance (%)*** | **96** | **90** | **96** |
| ***Side Effects*** | **Pain that disturbs sleep** | **Mild irritation, burning or redness** | **Mild pain** |
| ***Appearance after treatment*** | **Noticeable but easy to cover scar** | **Discoloured or bleached skin around the treated area** | **Noticeable but easy to cover permanent scar** |
| ***Cost to you (£)*** | **150** | **300** | **0** |
| ***Which situation would you prefer?***  ***(Tick one box only)*** | ***SURGERY*** | ***CREAM*** | *CURRENT SITUATION* |

## Scenario 2

You are told that :

- The chance of complete clearance of your BCC will be 96% if you have asurgery and 90% if you apply the cream.
- You will experience moderate pain for a time following surgery and severe irritation, burning, redness or ulceration DURING the treatment period if you apply a cream.
- Following treatment you will have a slightly raised permanent scar if you have surgery and skin as normal if you apply the cream
- The cost to you would be £150 for the surgery and £300 for the cream.

Summary

|  | ***SURGERY*** | ***CREAM*** | ***CURRENT SITUATION*** |
| --- | --- | --- | --- |
| ***Chance of complete clearance (%)*** | **96** | **90** | **96** |
| ***Side Effects*** | **Moderate pain** | **Severe irritation, burning, redness or ulceration** | **Mild pain** |
| ***Appearance after treatment*** | **Slightly raised permanent scar** | **Skin as normal** | **Noticeable but easy to cover permanent scar** |
| ***Cost to you (£)*** | **150** | **300** | **0** |
| ***Which situation would you prefer?***  ***(Tick one box only)*** | ***SURGERY*** | ***CREAM*** | *CURRENT SITUATION* |

## Scenario 3

You are told that :

- The chance of complete clearance of your BCC will be 94% if you have a surgery and 70% if you apply the cream.
- You will experience pain that disturbs sleep for a time following surgery and mild irritation, burning or redness DURING the treatment period if you apply a cream.
- Following treatment you will have a slightly raised permanent scar if you have surgery and skin as normal if you apply the cream
- The cost to you would be £750 for the surgery and £0 for the cream.

Summary

|  | ***SURGERY*** | ***CREAM*** | ***CURRENT SITUATION*** |
| --- | --- | --- | --- |
| ***Chance of complete clearance (%)*** | **94** | **70** | **96** |
| ***Side Effects*** | **Pain that disturbs sleep** | **Mild irritation, burning or redness** | **Mild pain** |
| ***Appearance after treatment*** | **Slightly raised permanent scar** | **Skin as normal** | **Noticeable but easy to cover permanent scar** |
| ***Cost to you (£)*** | **750** | **0** | **0** |
| ***Which situation would you prefer?***  ***(Tick one box only)*** | ***SURGERY*** | ***CREAM*** | *CURRENT SITUATION* |

## Scenario 4

You are told that :

- The chance of complete clearance of your BCC will be 94% if you have a surgery and 70% if you apply the cream.
- You will experience pain that disturbs sleep for a time following surgery and mild irritation, burning and redness DURING the treatment period if you apply a cream.
- Following treatment you will have a noticeable but easy to cover scar if you have surgery and discoloured or bleached skin around the treated area if you apply the cream
- The cost to you would be £0 for the surgery and £150 for the cream.

Summary

|  | ***SURGERY*** | ***CREAM*** | ***CURRENT SITUATION*** |
| --- | --- | --- | --- |
| ***Chance of complete clearance (%)*** | **94** | **70** | **96** |
| ***Side Effects*** | **Pain that disturbs sleep** | **Mild irritation, burning or redness** | **Mild pain** |
| ***Appearance after treatment*** | **Noticeable but easy to cover scar** | **Discoloured or bleached skin around the treated area** | **Noticeable but easy to cover permanent scar** |
| ***Cost to you (£)*** | **0** | **150** | **0** |
| ***Which situation would you prefer?***  ***(Tick one box only)*** | ***SURGERY*** | ***CREAM*** | *CURRENT SITUATION* |

**Scenario 5**

You are told that :

- The chance of complete clearance of your BCC will be 94% if you have a surgery and 70% if you apply the cream.
- You will experience pain that disturbs sleep for a time following surgery and mild irritation, burning or redness DURING the treatment period if you apply a cream.
- Following treatment you will have a noticeable but easy to cover scar if you have surgery and discoloured or bleached skin around the treated area if you apply the cream
- The cost to you would be £500 for the surgery and £750 for the cream.

Summary

|  | ***SURGERY*** | ***CREAM*** | ***CURRENT SITUATION*** |
| --- | --- | --- | --- |
| ***Chance of complete clearance (%)*** | **94** | **70** | **96** |
| ***Side Effects*** | **Pain that disturbs sleep** | **Mild irritation, burning or redness** | **Mild pain** |
| ***Appearance after treatment*** | **Noticeable but easy to cover scar** | **Discoloured or bleached skin around the treated area** | **Noticeable but easy to cover permanent scar** |
| ***Cost to you (£)*** | **500** | **750** | **0** |
| ***Which situation would you prefer?***  ***(Tick one box only)*** | ***SURGERY*** | ***CREAM*** | *CURRENT SITUATION* |

## Scenario 6

You are told that :

- The chance of complete clearance of your BCC will be 94% if you have a surgery and 70% if you apply the cream.
- You will experience moderate pain for a time following surgery and severe irritation, burning, redness or ulceration DURING the treatment period if you apply a cream.
- Following treatment you will have a barely visible permanent scar if you have surgery and slight lightening of skin around treated area if you apply the cream
- The cost to you would be £300 for the surgery and £500 for the cream.

Summary

|  | ***SURGERY*** | ***CREAM*** | ***CURRENT SITUATION*** |
| --- | --- | --- | --- |
| ***Chance of complete clearance (%)*** | **94** | **70** | **96** |
| ***Side Effects*** | **Moderate pain** | **Severe irritation, burning, redness or ulceration** | **Mild pain** |
| ***Appearance after treatment*** | **Barely visible permanent scar** | **Slight lightening of skin around treated area** | **Noticeable but easy to cover permanent scar** |
| ***Cost to you (£)*** | **300** | **500** | **0** |
| ***Which situation would you prefer?***  ***(Tick one box only)*** | ***SURGERY*** | ***CREAM*** | *CURRENT SITUATION* |

## Scenario 7

You are told that :

- The chance of complete clearance of your BCC will be 98% if you have a surgery and 50% if you apply the cream.
- You will experience moderate pain for a time following surgery and severe irritation, burning, redness or ulceration DURING the treatment period if you apply a cream.
- Following treatment you will have a noticeable but easy to cover scar if you have surgery and discoloured or bleached skin around the treated area if you apply the cream
- The cost to you would be £0 for the surgery and £150 for the cream.

Summary

|  | ***SURGERY*** | ***CREAM*** | ***CURRENT SITUATION*** |
| --- | --- | --- | --- |
| ***Chance of complete clearance (%)*** | **98** | **50** | **96** |
| ***Side Effects*** | **Moderate pain** | **Severe irritation, burning, redness or ulceration** | **Mild pain** |
| ***Appearance after treatment*** | **Noticeable but easy to cover scar** | **Discoloured or bleached skin around the treated area** | **Noticeable but easy to cover permanent scar** |
| ***Cost to you (£)*** | **0** | **150** | **0** |
| ***Which situation would you prefer?***  ***(Tick one box only)*** | ***SURGERY*** | ***CREAM*** | *CURRENT SITUATION* |

## Scenario 8

You are told that :

- The chance of complete clearance of your BCC will be 98% if you have a surgery and 50% if you apply the cream.
- You will experience mild pain for a time following surgery and moderate irritation, burning, redness or weeping DURING the treatment period if you apply a cream.
- Following treatment you will have a noticeable but easy to cover scar if you have surgery and severe irritation, burning, redness or ulceration if you apply the cream
- The cost to you would be £500 for the surgery and £750 for the cream.

Summary

|  | ***SURGERY*** | ***CREAM*** | ***CURRENT SITUATION*** |
| --- | --- | --- | --- |
| ***Chance of complete clearance (%)*** | **98** | **50** | **96** |
| ***Side Effects*** | **Mild pain** | **Moderate irritation, burning, redness or weeping** | **Mild pain** |
| ***Appearance after treatment*** | **Noticeable but easy to cover scar** | **Severe irritation, burning, redness or ulceration** | **Noticeable but easy to cover permanent scar** |
| ***Cost to you (£)*** | **500** | **750** | **0** |
| ***Which situation would you prefer?***  ***(Tick one box only)*** | ***SURGERY*** | ***CREAM*** | *CURRENT SITUATION* |

## Scenario 9

You are told that :

- The chance of complete clearance of your BCC will be 96% if you have a surgery and 90% if you apply the cream.
- You will experience mild pain for a time following surgery and moderate irritation, burning, redness or weeping DURING the treatment period if you apply a cream.
- Following treatment you will have a noticeable but easy to cover scar if you have surgery and discoloured or bleached skin around the treated area if you apply the cream
- The cost to you would be £500 for the surgery and £750 for the cream.

Summary

|  | ***SURGERY*** | ***CREAM*** | ***CURRENT SITUATION*** |
| --- | --- | --- | --- |
| ***Chance of complete clearance (%)*** | **96** | **90** | **96** |
| ***Side Effects*** | **Mild pain** | **Moderate irritation, burning, redness or weeping** | **Mild pain** |
| ***Appearance after treatment*** | **Noticeable but easy to cover scar** | **Discoloured or bleached skin around the treated area** | **Noticeable but easy to cover permanent scar** |
| ***Cost to you (£)*** | **500** | **750** | **0** |
| ***Which situation would you prefer?***  ***(Tick one box only)*** | ***SURGERY*** | ***CREAM*** | *CURRENT SITUATION* |

## Scenario 10

You are told that :

- The chance of complete clearance of your BCC will be 98% if you have a surgery and 50% if you apply the cream.
- You will experience mild pain for a time following surgery and moderate irritation, burning, redness or weeping DURING the treatment period if you apply a cream.
- Following treatment you will have a barely visible permanent scar if you have surgery and slight lightening of skin around treated area if you apply the cream
- The cost to you would be £750 for the surgery and £0 for the cream.

Summary

|  | ***SURGERY*** | ***CREAM*** | ***CURRENT SITUATION*** |
| --- | --- | --- | --- |
| ***Chance of complete clearance (%)*** | **98** | **50** | **96** |
| ***Side Effects*** | **Mild pain** | **Moderate irritation, burning, redness or weeping** | **Mild pain** |
| ***Appearance after treatment*** | **Barely visible permanent scar** | **Slight lightening of skin around treated area** | **Noticeable but easy to cover permanent scar** |
| ***Cost to you (£)*** | **750** | **0** | **0** |
| ***Which situation would you prefer?***  ***(Tick one box only)*** | ***SURGERY*** | ***CREAM*** | *CURRENT SITUATION* |

## Scenario 11

You are told that :

- The chance of complete clearance of your BCC will be 98% if you have a surgery and 50% if you apply the cream.
- You will experience moderate pain for a time following surgery and severe irritation, burning, redness or ulceration DURING the treatment period if you apply a cream.
- Following treatment you will have a slightly raised permanent scar if you have surgery and skin as normal if you apply the cream
- The cost to you would be £500 for the surgery and £750 for the cream.

Summary

|  | ***SURGERY*** | ***CREAM*** | ***CURRENT SITUATION*** |
| --- | --- | --- | --- |
| ***Chance of complete clearance (%)*** | **98** | **50** | **96** |
| ***Side Effects*** | **Moderate pain** | **Severe irritation, burning, redness or ulceration** | **Mild pain** |
| ***Appearance after treatment*** | **Slightly raised permanent scar** | **Skin as normal** | **Noticeable but easy to cover permanent scar** |
| ***Cost to you (£)*** | **500** | **750** | **0** |
| ***Which situation would you prefer?***  ***(Tick one box only)*** | ***SURGERY*** | ***CREAM*** | *CURRENT SITUATION* |

## Scenario 12

You are told that :

- The chance of complete clearance of your BCC will be 98% if you have a surgery and 50% if you apply the cream.
- You will experience pain that disturbs sleep for a time following surgery and mild irritation, burning or redness DURING the treatment period if you apply a cream.
- Following treatment you will have a noticeable but easy to cover scar if you have surgery and discoloured or bleached skin around the treated area if you apply the cream
- The cost to you would be £0 for the surgery and £150 for the cream.

Summary

|  | ***SURGERY*** | ***CREAM*** | ***CURRENT SITUATION*** |
| --- | --- | --- | --- |
| ***Chance of complete clearance (%)*** | **98** | **50** | **96** |
| ***Side Effects*** | **Pain that disturbs sleep** | **Mild irritation, burning or redness** | **Mild pain** |
| ***Appearance after treatment*** | **Noticeable but easy to cover scar** | **Discoloured or bleached skin around the treated area** | **Noticeable but easy to cover permanent scar** |
| ***Cost to you (£)*** | **0** | **150** | **0** |
| ***Which situation would you prefer?***  ***(Tick one box only)*** | ***SURGERY*** | ***CREAM*** | *CURRENT SITUATION* |

**Scenario 13**

You are told that :

- The chance of complete clearance of your BCC will be 98% if you have a surgery and 50% if you apply the cream.
- You will experience mild pain for a time following surgery and moderate irritation, burning, redness or weeping DURING the treatment period if you apply a cream.
- Following treatment you will have a slightly raised permanent scar if you have surgery and skin as normal if you apply the cream
- The cost to you would be £750 for the surgery and £0 for the cream.

Summary

|  | ***SURGERY*** | | ***CREAM*** | ***CURRENT SITUATION*** |  |
| --- | --- | --- | --- | --- | --- |
| ***Chance of complete clearance (%)*** | **98** | **50** | | **96** | |
| ***Side Effects*** | **Mild pain** | **Moderate irritation, burning, redness or weeping** | | **Mild pain** | |
| ***Appearance after treatment*** | **Slightly raised permanent scar** | **Skin as normal** | | **Noticeable but easy to cover permanent scar** | |
| ***Cost to you (£)*** | **750** | **0** | | **0** | |
| ***Which situation would you prefer?***  ***(Tick one box only)*** | ***SURGERY*** | ***CREAM*** | | *CURRENT SITUATION* | |

## Scenario 14

You are told that :

- The chance of complete clearance of your BCC will be 94% if you have a surgery and 70% if you apply the cream.
- You will experience moderate pain for a time following surgery and severe irritation, burning, redness or ulceration DURING the treatment period if you apply a cream.
- Following treatment you will have a barely visible permanent scar if you have surgery and slight lightening of skin around treated area if you apply the cream
- The cost to you would be £500 for the surgery and £750 for the cream.

Summary

|  | ***SURGERY*** | ***CREAM*** | ***CURRENT SITUATION*** |
| --- | --- | --- | --- |
| ***Chance of complete clearance (%)*** | **94** | **70** | **96** |
| ***Side Effects*** | **Moderate pain** | **Severe irritation, burning, redness or ulceration** | **Mild pain** |
| ***Appearance after treatment*** | **Barely visible permanent scar** | **Slight lightening of skin around treated area** | **Noticeable but easy to cover permanent scar** |
| ***Cost to you (£)*** | **500** | **750** | **0** |
| ***Which situation would you prefer?***  ***(Tick one box only)*** | ***SURGERY*** | ***CREAM*** | *CURRENT SITUATION* |

## Scenario 15

You are told that :

- The chance of complete clearance of your BCC will be 94% if you have a surgery and 70% if you apply the cream.
- You will experience pain that disturbs sleep for a time following surgery and mild irritation, burning or redness DURING the treatment period if you apply a cream.
- Following treatment you will have a barely visible permanent scar if you have surgery and slight lightening of skin around treated area if you apply the cream
- The cost to you would be £500 for the surgery and £750 for the cream.

Summary

|  | ***SURGERY*** | ***CREAM*** | ***CURRENT SITUATION*** |
| --- | --- | --- | --- |
| ***Chance of complete clearance (%)*** | **94** | **70** | **96** |
| ***Side Effects*** | **Pain that disturbs sleep** | **Mild irritation, burning or redness** | **Mild pain** |
| ***Appearance after treatment*** | **Barely visible permanent scar** | **Slight lightening of skin around treated area** | **Noticeable but easy to cover permanent scar** |
| ***Cost to you (£)*** | **500** | **750** | **0** |
| ***Which situation would you prefer?***  ***(Tick one box only)*** | ***SURGERY*** | ***CREAM*** | *CURRENT SITUATION* |

## Scenario 16

You are told that :

- The chance of complete clearance of your BCC will be 98% if you have a surgery and 70% if you apply the cream.
- You will experience moderate pain for a time following surgery and severe irritation, burning, redness or ulceration DURING the treatment period if you apply a cream.
- Following treatment you will have a slightly raised permanent scar if you have surgery and skin as normal if you apply the cream
- The cost to you would be £300 for the surgery and £500 for the cream.

Summary

|  | ***SURGERY*** | ***CREAM*** | ***CURRENT SITUATION*** |
| --- | --- | --- | --- |
| ***Chance of complete clearance (%)*** | **98** | **70** | **96** |
| ***Side Effects*** | **Moderate pain** | **Severe irritation, burning, redness or ulceration** | **Mild pain** |
| ***Appearance after treatment*** | **Slightly raised permanent scar** | **Skin as normal** | **Noticeable but easy to cover permanent scar** |
| ***Cost to you (£)*** | **300** | **500** | **0** |
| ***Which situation would you prefer?***  ***(Tick one box only)*** | ***SURGERY*** | ***CREAM*** | *CURRENT SITUATION* |

On a scale from 1 to 5, please state how difficult or easy it was to make your choices for the previous scenarios (please circle a number)

Extremely Easy Extremely Difficult

**1 2 3 4 5**

# *We would like to thank you for answering these questions. Your time is greatly appreciated.*
